# Critical care ultrasound training: a survey exploring the “education gap” between potential and reality in Canada

**DOI:** 10.1186/s13089-021-00249-z

**Published:** 2021-12-11

**Authors:** Jocelyn M. Slemko, Vijay J. Daniels, Sean M. Bagshaw, Irene W. Y. Ma, Peter G. Brindley, Brian M. Buchanan

**Affiliations:** 1 Department of Critical Care Medicine, Faculty of Medicine and Dentistry, University of Alberta, 2-124 Clinical Sciences Building, 8440-112 Street, Edmonton, AB T6G 2G3 Canada; 2 Division of General Internal Medicine, Faculty of Medicine and Dentistry, University of Alberta, 5-112 Clinical Sciences Building, 8440-112 Street, Edmonton, AB T6G 2G3 Canada; 3 Division of General Internal Medicine, Department of Medicine, University of Calgary, Foothills Medical Centre, North Tower, 9th Floor, 1403 – 29th Street NW, Calgary, AB T2N 2T9 Canada

**Keywords:** Ultrasonography, Echocardiography, Critical care, Intensive care, Medical education

## Abstract

**Background:**

Critical care ultrasound (CCUS) is now a core competency for Canadian critical care medicine (CCM) physicians, but little is known about what education is delivered, how competence is assessed, and what challenges exist. We evaluated the Canadian CCUS education landscape and compared it against published recommendations.

**Methods:**

A 23-item survey was developed and incorporated a literature review, national recommendations, and expert input. It was sent in the spring of 2019 to all 13 Canadian Adult CCM training programs via their respective program directors. Three months were allowed for data collection and descriptive statistics were compiled.

**Results:**

Eleven of 13 (85%) programs responded, of which only 7/11 (64%) followed national recommendations. Curricula differed, as did how education was delivered: 8/11 (72%) used hands-on training; 7/11 (64%) used educational rounds; 5/11 (45%) used image interpretation sessions, and 5/11 (45%) used scan-based feedback. All 11 employed academic half-days, but only 7/11 (64%) used experience gained during clinical service. Only 2/11 (18%) delivered multiday courses, and 2/11 (18%) had mandatory ultrasound rotations. Most programs had only 1 or 2 local CCUS expert-champions, and only 4/11 (36%) assessed learner competency. Common barriers included educators receiving insufficient time and/or support.

**Conclusions:**

Our national survey is the first in Canada to explore CCUS education in critical care. It suggests that while CCUS education is rapidly developing, gaps persist. These include variation in curriculum and delivery, insufficient access to experts, and support for educators.

**Supplementary Information:**

The online version contains supplementary material available at 10.1186/s13089-021-00249-z.

## Background

Critical care ultrasound (CCUS) allows physicians to rapidly diagnose and treat patients with a myriad life-threatening conditions [1–4]. It also facilitates real-time monitoring and greater procedural safety [1, 5–13]. Accordingly, it is an increasingly useful skill that needs to be taught, assessed and maintained: a job that typically falls on educators and ultrasound champions [14].

Experts have published recommendations [15, 16] in an effort to standardize the curriculum, its delivery, and how we assess competence in critical care ultrasound. While there are still unanswered questions about the best methods to implement and evaluate CCUS curriculum, the recommendations do provide a clear starting point and an instructive guide for those educators looking to formalize CCUS education. Unfortunately, little is known about the state of CCUS education in Canadian critical care residency training programs. There is reason to underscore this point; European and American surveys suggest a lack of trained faculty, and/or formal curriculum, and/or time to supervise trainees [2, 17]. This is the first nationwide Canadian survey examining whether national recommendations have been adopted, how CCUS education is delivered, how competence is assessed, and the challenges faced by educators and learners.

## Methods

We developed a 23-item survey and contacted all 13 Canadian centers with residency training programs in Adult Critical Care Medicine. Only one survey was collected per program, and we did not incentivize participation.

The survey was refined over several iterations. We started with a MEDLINE literature search using the MeSH terms "critical care", "ultrasound", "curriculum", "fellowship” and "medical education", and incorporated any manuscript that included curriculum content, design of objectives, educational strategies, methods of assessment and/or feedback, and perceived barriers [2, 16, 18]. These findings were merged with the Canadian CCUS recommendations^16^ to identify/describe major domains. Two national CCUS experts provided additional data, wording, clarity and content validity. The revised survey was reviewed by two additional physician-experts in CCUS (who did not participate in the study). Suggestions from the pilot were incorporated into the final survey, which is available in the Additional file 1: appendix online.

Surveys were integrated into an online platform (SurveyMonkey Inc. San Mateo, California, USA; www.surveymonkey.com) and disseminated via email with four reminders, 2 weeks apart, between January and March 2019. Survey results were compiled, and descriptive statistics presented.

Ethics approval was obtained from the University of Alberta Research Ethics Board (ref # Pro000086823). All participants consented.

## Results

Of 13 Canadian critical care training programs, 11 responded (85%). 6/11 (55%) respondents were program directors, and 5/11 (45%) were delegated local CCUS or curriculum champions. 10/11 respondents reported awareness of the Canadian CCUS training recommendations; 7/11 programs reported following them “closely” (64%), and 3/11 reported following them “somewhat”.

Table 1 summarizes CCUS curricular content and delivery. Critical care echocardiography was taught in 10/11 (91%) programs; lung and pleural space ultrasound was taught in 9/11 (82%) programs, but only 3/11 (27%, or less than one-third) taught how to assess for deep venous thrombosis (DVT). Only 4/11 programs (36%; approximately one-third) reported formal competency-based objectives, with entrustable professional activities and milestones.

**Table 1:** Development of current curricular delivery mechanisms and CCUS content

Delivery method	Number of programs		
	Fully developed^a^	In development^a^	Not yet developed^a^
Competency-based objectives	4 (36%)	3 (27%)	4 (36%)
Dedicated hands-on training	8 (73%)	3 (27%)	0
Dedicated image interpretation sessions	5 (45%)	3 (27%)	3 (27%)
Assessment of image acquisition skills	4 (36%)	1 (9%)	6 (55%)
Assessment of image interpretation	4 (36%)	1 (9%)	6 (55%)
Feedback mechanisms	5 (45%)	2 (18%)	4 (36%)
Quality assurance/case rounds	4 (36%)	1 (9%)	6 (55%)
Educational rounds	7 (64%)	2 (18%)	2 (18%)
Curriculum evaluation	3 (27%)	1 (9%)	7 (64%)

CCUS curricular content	Number of programs
Critical care echocardiography^b^	10 (91%)
Vascular access^b^	9 (82%)
Lung and pleural space^b^	9 (82%)
Abdominal free fluid^b^	7 (64%)
Renal ultrasound	4 (36%)
DVT assessment	3 (27%)

^a^Where fully developed indicates a no further work is required to implement and evaluate this aspect of the curriculum, and in development indicates that some deficiencies may still exist

^b^Denotes core competencies outlined by the Canadian recommendations

Curriculum delivery also differed. For example, while 8/11 (73%) used hands-on training, and 7/11 (64%) used educational rounds, only 5/11 (45%; less than half) incorporated routine feedback and almost two-thirds (7/11; 64%) did not formally assess skills in imaging acquisition or interpretation. Table 2 demonstrates the array of training strategies used, with a majority using textbooks (9/11: 82%), simulators (8/11: 73%) and websites (8/11: 73%). All 11 used at least one academic half day per annum, and approximately two-thirds relied upon unsupervised clinical service (7/11: 64%). In contrast, it was rare for programs to mandate a dedicated ultrasound rotation (4/11; 36%) or ultrasound course (2/11; 18%).

**Table 2:** Educational methods and materials in use for curricular delivery

Educational materials	
Textbooks	9 (82%)
Training simulator	8 (73%)
Websites	8 (73%)
Locally produced E-learning	7 (64%)
ICCU (CAE)© E-learning	6 (55%)
Sonosim® interactive learning	0
No additional resources provided	2 (18%)

Educational methods	Mandatory	Elective	Not available
Academic half-days	11 (100%)	0	0
As part of ICU service	7 (64%)	4 (36%)	0
Weekend/multiday courses	2 (18%)	6 (60%)	2 (20%)
Dedicated cardiology-based echo rotation	2 (18%)	7 (64%)	2 (18%)
Dedicated ICU-based US rotation	2 (18%)	3 (27%)	6 (55%)
Dedicated radiology-based US rotation	0	4 (36%)	7 (64%)

Subspecialty collaboration	
Cardiology	6 (55%)
Anesthesia	6 (55%)
Emergency medicine	3 (30%)
Internal medicine	1 (10%)
Radiology	1 (10%)

The amount of dedicated hands-on training with an instructor (outside of clinical care) also varied. For example, 1 program reported delivering 1 to 4 h, whereas 3 programs providing greater than 15 h. Regarding contributions from other specialties, cardiology (6/11; 55%) and anesthesia (6/11; 55%) taught disproportionately compared to emergency medicine, internal medicine and radiology.

Table 3 summarizes potential barriers. On the positive side, regarding access to US machines, 10/11 (91%) programs reported “no barrier” to machines, and an average of 2 machines per 20–30 beds. In contrast, regarding access to experts, only 4 programs (36%; approximately one-third) felt it was “easy” to get local training, with 5 reporting “mild difficulty”, and 2 reporting “marked difficulty”. Overall, the most common “critical” or “major” barrier was difficulty identifying a local expert (4/11: 36%), inadequate supervision (7/11: 64%), and inadequate academic support (4/11; 36%). 9/11 programs identified having only 1 or 2 local experts (range 0–6).

**Table 3:** Barriers identified to be hindering curricular development and implementation

	Critical barrier	Major barrier	Minor barrier	No barrier
Lack of time for an educator	3 (27%)	4 (36%)	3 (27%)	1 (9%)
Lack of academic support	3 (27%)	1 (9%)	6 (55%)	1 (9%)
Difficulty identifying a local expert	1 (9%)	3 (27%)	3 (27%)	4 (36%)
Inadequate ability to supervise	1 (9%)	6 (55%)	1 (9%)	3 (27%)
Collaboration with others	1 (9%)	1 (9%)	3 (27%)	6 (55%)
Lack of formal curriculum	0	2 (18%)	4 (36%)	5 (45%)
Lack of fellow time	0	1 (9%)	5 (45%)	5 (45%)
Number of scans required	0	1 (9%)	4 (36%)	6 (55%)
Lack of equipment	0	1 (9%)	0	10 (91%)

The minimum training requirements and typical methods of assessment were also explored. Approximately one-third of programs (4/11: 33%) required fellows to perform and interpret a minimum number of CCUS exams. All 4 programs required a minimum number of echocardiograms. 75% required a minimum number of lung and pleural ultrasounds and 25% required a minimum number of thoracenteses, paracenteses, and abdominal free fluid and vascular access scans. Approximately half of the programs used portfolio review, one program used a written formal exam, and three programs implemented an objective structured clinical exam (OSCE). Feedback was most often delivered in real time from a local expert (7/11: 64%), but also remotely with the use of USB image storage and digital archiving software (4/11, 36% each). Quality assurance rounds were only performed in two programs. Additional competency assessment tools included entrustability assessment, in-training evaluation reports (ITERs), CAE-ICCU© modules, online modules, and reliance on the National Board of Echocardiography CCM exam. 7/11 programs assessed learner experiences using feedback surveys, exams, and/or local research, whereas 4/11 did not evaluate learners.

## Discussion

This is the first national evaluation of the Canadian CCUS education in critical care and summarizes the “educational gap” between recommendations and local educational practices, as shown in Table 4 [16].

**Table 4:** Comparison of Canadian CCUS recommendations [16] to the current state

Canadian recommendations for critical care ultrasound training and competency	Survey results
Academic centers Commitment to create and sustain a local CCUS program One machine per unit dedicated to CCUS	Lack of equipment was not a barrier in 91%
Local experts Support to sustain and/or train local CCUS expert(s) experienced in general CCUS and basic critical care echo Be supported with time and funding Support for faculty development if no local expert exists	64% feel identifying a local expert as a barrier but 82% has 1–2 per training site Lack of time for educator a barrier in all programs, lack of academic support a barrier in 82%
Curriculum implementation Didactic and hands-on training in general CCUS and basic echo (10 h each) Core applications that should be taught: basic critical care echo, lung/pleura, guidance of vascular access, identification of free fluid Optional applications include DVT diagnosis, renal ultrasound and abdominal aorta	Hours of dedicated hands-on training: 36% 5–9 h and 27% 10–15 h All programs have formal teaching basic critical care echo 82% have formal teaching in lung/pleural space, 82% vascular access, 64% abdominal free fluid 27% formally teaching DVT, 33% renal
Portfolio building Supervised studies in core exam types with feedback Performed on patients over simulators Minimum number of studies required in core applications Portfolio kept of completed scans Feedback/supervision should be in real time with local expert at bedside, or through digital storage	Inability to supervise a barrier in 55% 64% of programs do not have a minimum number of studies required 73% are using a training simulator 50% use portfolio review 45% have fully developed feedback mechanisms, 64% receive feedback in real time at the bedside, 36% USB and/or digital archive
Assessment of competency Each learner should have a final assessment in image acquisition, interpretation, and clinical integration Method for continuing competence: image review sessions, lectures, etc.	44% have dedicated assessment for trainees 11% formal written exam, 33% OSCE

Our data highlight encouraging signs but important caveats. First, all responding sites teach basic critical care echocardiography, many teach pulmonary ultrasound, and access to machines does not appear to be a substantial issue. In contrast, few teach how to detect abdominal free fluid (64%), do DVT assessment (27%), or perform renal ultrasound (36%). Moreover, few mandate formal training; few formally assess image acquisition and interpretation skills, and educators still feel under-supported.

Canadian academic centers are well-resourced compared to most jurisdictions. Therefore, it is noteworthy that cultural barriers persist. Moreover, these concerns are not unique or new. In a 2014 survey of American CCM fellowship directors, many reported insufficient experts to teach and supervise, and not enough faculty who modeled the use of ultrasound themselves [2]. Similarly, a 2017 survey of intensive care societies in Western European countries reported insufficient time, trainers, and consensus regarding core competencies [17]. Our work further highlights the importance of in-house educators, champions, mentors, supervision, and regular program evaluation.

Our results highlight that it is still rare for trainees to undertake dedicated CCUS rotations or for educators to receive protected time. Moreover, less than half of our programs assessed CCUS competency. National CCUS recommendations [16], highlight the need for deliberate assessment, and include ideas such as a portfolio and hands-on exam. This need not be onerous. After all, images can now be easily logged on a USB drive, or on the ultrasound machine, or via digital archiving systems. Our results also highlight that programs do not routinely evaluate curricula. Creating national groups, such as the Canadian Internal Medicine Ultrasound Group, could help with standardization and resource sharing [19].

Our study has limitations. For example, it is hard to objectively define what constitutes “good” instruction or supervision, just as it is difficult to state when a curriculum is “optimal” or “mature”; indeed, there are not clear published “best practice” standards for CCUS education. Further, Canadian recommendations [16] are expert panel-driven, and do not include a robust methodology. Nonetheless, in the absence of competing Canadian recommendations, we felt that this panel of experts from across Canada provided an informed and representative starting point and that mirrors those in the United States [2] and Europe [15]. We also relied upon assessments by program directors or champions rather than the opinion of the end-user, i.e., the trainee. Canada only has thirteen Adult Critical Care training programs, limiting our sample size. Lacking responses from only two programs may skew our results. We also focused on academic centers and were unable to capture the benefits gained through informal instruction or self-teaching. Despite these limitations, our acceptable response rate (85%), and a survey that covered multiple domains (e.g., content, delivery, infrastructure, and barriers) shows that CCUS is increasingly seen as an important in CCM training. Accordingly, we need to support both trainees and educators, and to close the gap between potential and reality.

Finally, one area in which this study did not explicitly examine is the evolving role of formal certification in CCUS in Canada. Over the last couple of years as critical care medicine has transitioned to competency-based medical education in Canada, key CCUS modalities (i.e., heart, lung, abdomen, vascular) have been selected as “required training experiences” for the sub-specialty of Critical Care Medicine by the Royal College Objective of Physicians and Surgeons of Canada [20]. Therefore, measures of assessment including Entrustable Professional Activities and licensing examinations do systematically assess these competencies in light of national standards for achievement. In contrast, the National Board of Echocardiography in the United States now provides a separate certification pathway through the “Examination of special competence in critical care echocardiography (CCEeXAM)” [21] for more advanced ultrasound examinations, which began in 2019. In our survey, only one training program highlighted that this was a part of their assessment strategy. While this exam may play a role in future certification in Canada, it has yet to be adopted by any national licensing body.

## Conclusions

Critical care ultrasound is a useful adjunct in caring for the critically ill patient; ensuring that future critical care practitioners can perform this skill set is necessary. Our findings show that disparities in CCUS education persist and that more work needs to be done to achieve standardization of CCUS education across Canada. Expert recommendations have served as a practical benchmark in this study, but we acknowledge that there may be debate whether such recommendations constitute “best practice” evidence to inform curriculum design. Practical rotation-based exposure mandated by programs and formal assessment of technical skill and knowledge may be limited. Further, trained faculty, access to local champions, and dedicated time are scarce. Through program collaboration and prioritization of CCUS training for both faculty and trainees, closing the gap between recommendations to educational practice could easily be achieved.

## Supplementary Information

**Additional file 1.** Survey—Residency program director or delegate.

## Data Availability

The datasets used and analyzed during the current study are available from the corresponding author on reasonable request.

## Abbreviations

CCUS: Critical care ultrasound
CCM: Critical care medicine
ITERs: In-training evaluation reports
