# Supplementary material for: Critical care ultrasound training: a survey exploring the “education gap” between potential and reality in Canada
Source: Ultrasound J. 2021 Dec 11;13:48. doi: 10.1186/s13089-021-00249-z (PMC8665911; doi:10.1186/s13089-021-00249-z)
Supplement: Supplementary file 1 — Additional file 1. Survey—Residency program director or delegate. [file 13089_2021_249_MOESM1_ESM.docx]

**Survey – Residency program director or delegate**

The following survey will address the current state of critical care ultrasound training at your academic institution. Your response will not be tied to your institutional affiliation and your response will remain anonymous. This survey will help us understand the disparity between ideal and existing conditions in national CCUS training in critical care medicine.**Although we are asking you to record what CCM training program you work with, this data will remain confidential, is only for statistical purposes and will not be reported in any publication.**

**Current Curriculum**

**______________________________________________________________________________1. Which CCM training program do you currently work with?**

1. Dalhousie University
2. Université de Montréal
3. McGill University
4. Université Laval
5. University of Toronto
6. McMaster University
7. Western University
8. University of Ottawa
9. Queens University
10. University of Manitoba
11. University of Alberta
12. University of Calgary
13. University of British Columbia

1. **What is your current role with critical care ultrasound training for sub-specialty critical care medicine trainees?**
2. Program director
3. Delegated individual (e.g. ultrasound champion, curriculum lead)
4. Other: (please specify):

1. **How often is bedside ultrasound being employed by fellows in your critical care units for indications other than central vascular access?**

| Never | Rarely (once per month or less) | At least once per week | Multiple times per week (but not necessarily daily) | At least once daily | Multiple times per day |
| --- | --- | --- | --- | --- | --- |

1. **How many sub-specialty critical care medicine trainees does your affiliated program have?**
2. 0-5
3. 5-10
4. 10-15
5. 15-20

1. **A curriculum in critical care ultrasound can include everything from explicit objectives and dedicated hands-on training to assessment modalities and program evaluation. Can you please select all aspects that currently apply to your existing curriculum in critical care ultrasound for critical care medicine trainees?**

|  | Not yet | In-development | Fully Developed |
| --- | --- | --- | --- |
| Formal competency-based objectives (EPAs, milestones) |  |  |  |
| Dedicated hands-on training |  |  |  |
| Dedicated image-interpretation sessions for learning |  |  |  |
| Formal assessment of trainee image acquisition skills |  |  |  |
| Formal assessment of trainee image interpretation |  |  |  |
| Feedback mechanisms (ie. USB sticks, Qpath, etc) |  |  |  |
| Quality assurance/ case rounds to review studies (regularly scheduled) |  |  |  |
| Regularly scheduled educational rounds |  |  |  |
| Curriculum evaluation (internal review of curriculum) |  |  |  |
| Other (please specify) |  | | |

1. **In 2014, the Canadian recommendations for critical care ultrasound training and competency were published, prescribing a model for CCUS curriculum development in Canadian Critical Care Residency Training Programs. Are you aware of these recommendations?**
2. Yes
3. No

**(logic question) only if select yes above--🡪**

1. **With reference to the above recommendations in question 4, please describe if or how these recommendations have informed your training program?**
2. I don’t know
3. Not at all
4. Somewhat
5. Closely
6. Very closely

1. **Which components of CCUS are currently being taught formally in your residency training program (formal = dedicated, apart from clinical care, informal = integrated with clinical care)? (check all that apply)**

| **Modality** | **Yes, formal and informal teaching** | **Yes, informal teaching** | **No, not taught** |
| --- | --- | --- | --- |
| Basic trans-thoracic critical care echo (RV, LV, IVC function, pericardial effusion) |  |  |  |
| Basic trans-esophageal critical care echo (RV, LV, IVC function, pericardial effusion) |  |  |  |
| Advanced assessment of stroke volume and cardiac output |  |  |  |
| Advanced assessment of regurgitant valvular lesions |  |  |  |
| Lung and pleural space examination |  |  |  |
| Thoracentesis & procedural guidance |  |  |  |
| Vascular access & procedural guidance |  |  |  |
| Abdominal free fluid & procedural guidance |  |  |  |
| Assessment for deep venous thrombosis |  |  |  |
| Transcranial Doppler |  |  |  |
| Hydronephrosis and bladder assessment |  |  |  |
| Other: | Free text | | |

**Resources, infrastructure and barriers to curriculum development ______________________________________________________________________________**

1. **How many local intensivists, competent in both critical care echocardiography and/or general CCUS (i.e. focused lung/thoracic, focused abdominal), are accessible to your CCM training program per hospital site to facilitate CCUS training?**
2. 0 per training site
3. 1-2 per training site
4. 3-4 per training site
5. 5-6 per training site
6. >6 per training site

1. **How easily can a faculty intensivist at your institution gain skills in performance of CCUS (in at least basic applications) with *local resources? (including mentorship, access to experts, workshops, conferences)**
2. Not difficult (easy to obtain training locally)
3. Mild difficulty (limited resources to facilitate training, but still possible)
4. Severely difficult (uneasy to obtain; unlikely to happen)
5. I am unsure

1. **What is the average ratio of ultrasound machines to beds in all of your current intensive care units?**

_______ machines to ______  beds

1. Below are barriers to training fellows in CCUS that are identified in the literature. Please rate the degree of difficulty these barriers (or suggested barriers) has presented in development of your own curriculum? (Select one per row)

|  | **Not a barrier** | **Minor barrier** (i.e. minimal concern that has already been addressed with current resources) | **Major barrier**  (i.e. challenging but is currently being addressed with resources) | **Critical barrier** (i.e. unable to be addressed effectively with current resources) |
| --- | --- | --- | --- | --- |
| Difficulty identifying a local expert |  |  |  |  |
| Lack of overall faculty expertise |  |  |  |  |
| Lack of formalized curriculum |  |  |  |  |
| Inadequate ability to supervise due to faculty time constraints |  |  |  |  |
| Lack of time in fellow’s schedule to perform and interpret scans |  |  |  |  |
| The number of scans required for competence is too high |  |  |  |  |
| Lack of equipment (including poor quality equipment) |  |  |  |  |
| Difficult collaboration with other specialties (i.e. cardiology, radiology) |  |  |  |  |
| Other/additional barriers (s) |  |  |  |  |

**Training Methods ______________________________________________________________________________**

1. **How many hours of dedicated hands-on training (i.e. not solely while providing clinical care) with a designated instructor do your critical care medicine trainees receive during their sub-specialty training?**

1. 0 hours
2. 1-4 hours
3. 5-9 hours
4. 10-15 hours
5. > 15 or more

1. **What training materials or resources are provided/available to critical care medicine fellows? (select all that apply)**

- Locally produced E-learning solution (i.e. website, locally produced tutorials,etc)
- ICCU (CAE): E-learning based curriculum in CCUS
- Textbooks
- SonoSim interactive simulator training
- Website: ____________
- CAE Vimedix^TM^ training simulator
- MedaPhor Heartworks^TM^ /other training simulator
- Additional dedicated CCUS learning resources are not provided
- Other: (free text available)

1. **What methods of training do your critical care medicine fellows have the option of participating in? (select one per row)**

|  | Mandatory | Elective | Not available |
| --- | --- | --- | --- |
| Academic half days |  |  |  |
| Local weekend or multi-day courses |  |  |  |
| Dedicated cardiology-based Echo rotation |  |  |  |
| Dedicated radiology-based US rotation |  |  |  |
| Dedicated Intensive care-based US rotation |  |  |  |
| Dedicated emergency-medicine based US rotation |  |  |  |
| While fellow is on ICU service |  |  |  |
| Other (please specify) |  |  |  |

1. **Many critical care programs across Canada deliver their curriculum in conjunction with other specialties (i.e. especially cardiology for echo). Please select any or all of the following departments that assist in delivery of your curriculum: (check all that apply)**

|  | Yes | No |
| --- | --- | --- |
| Cardiology |  |  |
| Radiology |  |  |
| Emergency medicine |  |  |
| Anesthesia |  |  |
| Internal medicine |  |  |
| Other: |  |  |

**Assessment and monitoring of trainee performance ______________________________________________________________________________**

1. **Are residents required to perform and interpret a set number of scans for each system (e.g. 20 lung ultrasounds, 40 cardiac ultrasounds) during their critical care medicine fellowship?**

       Yes perform__      Yes, perform and interpret__       Neither__     Unsure __

1. **If yes, please enter the number of CCUS scans each fellow is required to complete in your current program (a blank is presumed as “not required”):**

- Critical care echo: (free text space)
- Lung/pleural: (free text space)
- Thoracentesis procedural guidance (free text space)
- Vascular Access: (free text space)
- Abdominal free fluid: (free text space)
- Paracentesis procedural guidance_
- Other: (free text space)

1. **Please specify if and how you currently evaluate a CCM trainee for achieving competency in CCUS, other than the Royal College final critical care medicine exam (Select all that apply):**

- Dedicated (or specific) CCUS assessment Yes__No__
- Portfolio review   Yes__ No__
- Formal CCUS exam (Written/computer-based)   Yes __ No__
- Objective structured clinical exam  Yes __ No__
- Other______

1. **Please describe any specific assessment of competency tools being utilized?**

(free text space)

**Curriculum evaluation activities ______________________________________________________________________________**

1. **There are a variety of methods of quality assurance to provide feedback on CCUS studies to learners, ranging from USB storage to more elaborate imaging storage software (i.e. Qpath). Which regularly reviewed methods currently apply to your CCUS curriculum? (select all that apply)**

- No routine standardized method to provide feedback yet_
- In real-time with local expert at the bedside_
- USB storage by individual learners for later review___
- Storage on the machine by individual learner for later review___
- Remotely, with digital archiving (i.e. Qpath), after the scan has been completed
- CCUS quality assurance rounds
- Other digital solution _______
- Other___

1. **Please described any method you are currently applying to monitor or study curricular effectiveness? (i.e. research, exams, etc)**

1. **Further comments (box) (“if you would like to make any further comments, please note them here)**
